# T.E.A.M.S.Work: Leveraging Technicians to Enhance ABM Med Sync in Community Pharmacies

**DOI:** 10.3390/pharmacy8020051

**Published:** 2020-03-27

**Authors:** Tamera D. Hughes, Lana M. Minshew, Stacey Cutrell, Stefanie P. Ferreri

**Affiliations:** UNC Eshelman School of Pharmacy, University of North Carolina, Chapel Hill, NC 27599, USAminshew@live.unc.edu (L.M.M.); scutrell@live.unc.edu (S.C.)

**Keywords:** medication synchronization, community pharmacy, pharmacy technicians, pharmacy workforce

## Abstract

The expansion of pharmacy technicians’ roles in community pharmacies allows pharmacists the opportunity to focus on providing clinical services to patients. This study explores the tasks pharmacy technicians’ perform to support Med Sync programs in community pharmacies. Pharmacy staff members at North Carolina pharmacies with more than fifty percent of their prescription volume being dispensed as part of a Med Sync program were recruited to participate in semi-structured interviews. Inductive coding and summary analysis were used to analyze the interview data. Study participants described pharmacy technicians’ roles in identifying patients for marketing and enrollment, reviewing patients’ medications list, choosing alignment dates based on patient preference, contacting patients in preparation for dispensing and, lastly, engaging in pickup or delivery of medications. This study highlights technicians’ vital role in completing tasks that support Med Sync programs in community pharmacies.

## 1. Introduction

In response to value-based payment structures, community pharmacies recognize the expansion of pharmacy technicians’ roles for achieving optimal patient care [1]. Both medication dispensing support and clinical service support have been adopted by pharmacy technicians [2]. Technicians’ roles have expanded to include taking and transferring prescriptions and “tech-check-tech” duties with no statistically significant differences detected in the accuracy or error-detection rates between pharmacists and technicians [3]. Advanced roles, such as, immunization administration have also emerged in some states to include technicians, further encouraging the advancement of their roles [1]. The evolution of technician roles better positions pharmacy technicians to support and free up pharmacists to focus on providing patient care services [4]. 

Medication nonadherence is estimated to account for nearly $300 billion of the annual healthcare cost in the United States [5]. In an effort to improve adherence and reduce unnecessary spending, medication synchronization (Med Sync) programs have been adopted by community pharmacies [6]. Studies show medication adherence improves when patients are enrolled in a Med Sync program; however, considerable variability in the implementation of this service exists between community pharmacies [7,8,9,10,11]. Med Sync, as described by the American Pharmacists’ Association (APhA) in their white paper, is designed to improve consumers’ adherence to medications and build efficiencies in pharmacy operations. The white paper establishes how community pharmacies can integrate the Appointment Based Model (ABM) Med Sync into pharmacy workflow and business models [12]. The 10 steps outlined in the white paper were summarized in a systematic review of the Med Sync process conducted by Patti and colleagues [12,13]. The systematic review revealed 5 core components: (1) pharmacy staff identifying and enrolling patients, (2) pharmacy staff reviewing and assessing medication, (3) pharmacy staff working with patients to synchronize medication refills, (4) pharmacy staff contacting patients or designated care providers to identify medications for fill, and (5) patients meeting with pharmacy staff for pick up or delivery of medication [13]. The white paper and the systematic review demonstrate key roles that must take place to perform Med Sync services, yet neither document mentions which pharmacy staff members should perform these services.

Across the country, community pharmacies have implemented Med Sync to promote medication adherence and improve patient outcomes. The National Community Pharmacists Association (NCPA) *Digest* reports 79% of independent pharmacies currently offer Med Sync to combat nonadherence [14]. Researchers identified several pharmacies in North Carolina who dispense more than fifty percent of their prescriptions as part of a Med Sync program. To explore how these community pharmacies operate Med Sync, a qualitative study was undertaken with the aim to reveal strategies that incorporate technicians’ roles into Med Sync. Determination of these roles and responsibilities will provide insight into specific pharmacy operations employing technicians for successfully operating a Med Sync program. 

## 2. Materials and Methods 

The research team consisted of two pharmacists, one pharmacy student, and a qualitative research methodologist. The study reported here is a part of a larger multi-phase project examining adoption of Med Sync programs in community pharmacies. For the purposes of the study reported here, the data that focuses on the role of pharmacy technicians is highlighted. This observational study utilized a semi-structured interview guide with the initial goal of identifying the barriers and facilitators to adoption and explored community pharmacies practical solutions to ensure successful adoption of Med Sync services. Purposeful sampling was used to identify North Carolina community pharmacies with greater than 50% of their prescriptions in a Med Sync program and the leads of the Med Sync program were invited to participate in an interview. Interviews were conducted by three members of the research team via Zoom Client for Meetings [computer program] Version 4.6.7. (Zoom Video Communications Inc., San Jose, CA, USA). The semi-structured interview guide focused on all aspects of adoption of Med Sync. Each interview lasted approximately 60 min and were transcribed verbatim. 

The analysis of interview data used an inductive approach to coding, and codes were derived from the data in order to reflect participants’ perspective [15,16]. As a group, the team read and discussed participant responses and through these discussions created codes and corresponding definitions. Memos were written during and after each coding session to capture the analytic process and any themes or patterns that were emerging in the data [15,16]. Preliminary analysis revealed heavy involvement of non-pharmacist staff, leading the research team to further investigate the roles and tasks of technicians in Med Sync. After initial coding, cluster analysis was used to focus on the data that emphasized the roles of and tasks completed by technicians [15,16]. This process involved creating a summary matrix of the data and reviewing the data iteratively to identify key ideas expressed by participants regarding the role and tasks of technicians in the Med Sync program. At least two members of the research team analyzed the qualitative data at a given time and agreed on the application of codes and the identified themes. A third researcher, a pharmacist, verified all themes. This study (IRB# 19-1832) was determined to be exempt by the university’s IRB. 

## 3. Results

Twelve community pharmacies met the inclusion criteria of having greater than 50% of their prescriptions in a Med Sync program and were invited to participate in the study. Seven pharmacies responded and agreed to be interviewed, Table 1 displays their demographic characteristics. The recruitment email requested to interview an individual who was the primary leader of the Med Sync program at the pharmacy. Six pharmacists and one pharmacy technician were interviewed. 

Analysis of the seven community pharmacies revealed technicians’ support of Med Sync through various roles and tasks. Participants described technicians’ roles in identifying patients for marketing and enrollment, reviewing patients’ medications list to establish a plan for synchronization, choosing alignment dates based on patient preference, contacting patients in preparation for dispensing, and lastly, engaging in pickup or delivery of medications. 

Program leaders from each store described varying levels of technician involvement in all aspects of the Med Sync Program. Each pharmacy recounted technician responsibilities in at least three of the tasks mentioned above. One pharmacy acknowledged technician involvement in all Med Sync tasks, and another pharmacy had a technician involved in four. All pharmacies detailed involvement in both documentation of patient information as part of the Med Sync process and preparation and packaging of prescriptions. Two pharmacies discussed technician involvement in addressing additional interventions, such as, delivery. Six of the seven pharmacies described technician assistance with patient enrollment. 

Tasks mentioned in the marketing and enrollment of Med Sync patients included identifying nonadherent patients via performance information management systems and communicating with new and frequent patients during face to face and telephonic encounters. For instance, one participant shared they have technicians “*run a report of everybody who is less than 80% adherent*” as a way to target patients for enrollment in Med Sync. Patient assessments and medication review tasks included technicians identifying low, medium, and high-risk patients for medication nonadherence based on medication burden and prior incidence with adherence. One participant, a pharmacist, when asked to “Walk me through how you synchronize your prescriptions once they are enrolled”, responded, “*That’s probably a better question for the techs.*” In addition, technicians also documented patient information, such as, counting refills and patient’s “at home stock”. For example, one participant stated that technicians “*tally up how many pills of each medication we need to give to the [patient] … and short fill whatever needs to be short filled to get [the patient] lined up*.” Technicians interacted with patients to set synchronization dates that accommodated finances, transportation and other patient limiting preferences. One participant noted their technicians set sync dates based upon patient preference, “*we leave it up to the individual [patient] … they tell us when [they] want it, what day or what week and we kind of go from there*.” During the preparation of the medications for dispensing, technicians conducted routine patient interviews and packaging of prescriptions using multi-dose packaging systems. Lastly, technicians were either present for pickup or addressed additional services, such as, delivery when necessary. 

Additional corresponding participant quotes capturing technician roles, responsibilities, and tasks are matched with the identified 5 major themes in Table 2.

## 4. Discussion

In this study, pharmacy technicians were identified as having varying assignments in Med Sync programs in community pharmacies who had 50% or more prescriptions in the program. Our results demonstrate that technicians can support Med Sync programs by marketing and enrolling patients, reviewing patient prescriptions, selecting medication synchronization dates, and assisting in the delivery or pick up of medications. These results help to close the gap on the conversation as to whether Med Sync services fall on pharmacists and initiate the capturing of non-pharmacist staff participation in the Med Sync process [13]. This study demonstrates that technicians can be engaged in all tasks of the Med Sync process. 

The APhA white paper establishes the steps to improve consumers’ adherence to medication and the systematic review conducted by Patti and colleagues summarizes the white paper to help standardize Med Sync within community pharmacies. [13,16]. The white paper and the systematic review demonstrate key roles that must take place to perform Med Sync services, yet neither document mentions which pharmacy staff members should perform these services. The five activities that technicians participated in that emerged from our data are reflective of the Patti and colleagues 5 core components. Participant responses alluded to technicians’ practicality in performing most, if not all tasks. This suggests that technicians are and can be integral components for effective Med Sync implementation.

According to the results, technicians have constant engagement with patients and are in a great position for involvement in all steps of a Med Sync program from initiation to pick up and/or delivery. In fact, one of the pharmacies mentioned technician involvement in all 5 core components of the Med Sync process, and the technician was the primary lead on the service. This demonstrates that technicians are capable of participating in all steps of the Med Sync process, and technicians are also in a great position to take the lead of the service. Recent studies have shown having a dedicated Med Sync technician assists in supporting clinicians [17,18]. In the current study, one participant, a pharmacist, deferred their question regarding patient synchronization and enrollment responding, “*That’s probably a better question for the techs.*” This suggests the pharmacist trusted the technician to be the leader of the Med Sync program. 

Not only are technicians in a great position to lead the program, they are also able to assume increased responsibility. Two recent workforce surveys suggest technician responsiveness and eagerness in assuming increased responsibility [1,19]. When the lone pharmacy technician was asked how they became involved in the program they responded, “*I have an eye for organization, and I just started taking it over, little by little*.” This represents the increased responsibility the technician was willing to take on to lead the program. In addition, to leading the Med Sync program, the technician also attended a national meeting to learn more about the service. This further solidified the technician’s commitment as a leader and their continued involvement in Med Sync. 

In other observations of pharmacies struggling to implement new services, underutilization of pharmacy technicians is a common theme. Given the challenging practice environment that community pharmacists are faced with, efficiently involving all staff members in Med Sync operation is key. Participant responses were consistent in leveraging the technician workforce in support of Med Sync success. Though this study focused on the role of technicians, future research needs to investigate the roles of additional non-pharmacist staff. Clerks and cashiers were mentioned in multiple steps in the Med Sync process. One participant acknowledged the importance of the cashiers in their Med Sync enrollment process stating “*cashiers... would get a lot more [enroll more patients] because they were talking face to face with people*”.

Finally, this study expands the literature regarding advancing the roles for pharmacy technicians in community pharmacies. By allowing technicians to have more advanced technical roles, it provides community pharmacist opportunities to become more involved in direct patient care. This transition allows pharmacists to participate in activities that use their expertise in medication optimization services and improves medication outcomes. Inevitably, advancing pharmacy practice depends on elevating the roles and responsibilities of pharmacy technicians. 

### Study Limitations

The current study focused solely on community pharmacies in North Carolina that had 50% or more of their prescriptions enrolled in a Med Sync program. More knowledge could be gained by broadening the participant sample to include community pharmacies in other states. Despite the small sample, the current study does include both urban and rural community pharmacies and a range of prescription volume per week indicating technicians can support Med Sync utilization in a variety of contexts. Furthermore, expanding this research to determine how technicians’ roles affect patients’ outcomes may be beneficial and is warranted. 

## 5. Conclusions

Effective leveraging of pharmacy technician roles is important to the success of Med Sync programs. This study highlights technicians’ ability to support Med Sync programs in community pharmacies. The roles of pharmacy technicians and other workforce personnel must continually expand in an attempt to meet the needs of an ever-changing healthcare landscape. Continuous advancements in the responsibilities of pharmacy technicians will undoubtedly advance community pharmacy practice. 

## Figures and Tables

**Table 1:** Participating Community Pharmacy Characteristics.

Characteristics	Exemplar 1	Exemplar 2	Exemplar 3	Exemplar 4	Exemplar 5	Exemplar 6	Exemplar 7
Geographic Region	Rural	Urban	Urban	Rural	Rural	Urban	Urban
Average Prescription volume per week	1750	900	650	4500	750	2000	250
Years of Reported MedSync Services	4–5	7	3	3-5	5	4	2

**Table 2:** Quotes Discussing Technician Activities in Medication Synchronization.

Activity—Major Themes	Participant Quote
**Market and Enroll** Creation of a structured system to target and enroll patients who are most likely to benefit from a medication synchronization program.	“And so, our analytics technician, she would go in and run a report of everybody who is less than 80% adherence.” (Exemplar 1) “Technicians market the program” (Exemplar 4) “I mean, it’s more of just like a technician says, hey, listen, this person isn’t on a Med Sync that they’d be a prime candidate.” (Exemplar 6) “If they’re on monthly medications…. the maintenance medications that they need to be on all the time…. we [the technicians identify] for our sync program.” (Exemplar 7)
**Medication review and patient assessment** Assessment and review of patients’ medication prior to synchronization.	“The technician who’s working sync that day, they’ll take those forms and then you know tally up you know how many pills of each med we need to give to the customer.” (Exemplar 1) “So, our categories are green, yellow, red, and those are what our technicians [review] …. And so sometimes it is like a pharmacist or technician referral … we kind of watch them a little closer make sure they’re getting what they need.” (Exemplar 6) “I’m [technician] the main one that does the initial contact and then the initial drop of the prescriptions… If there’s any issues, we let them know.” (Exemplar 7)
**Align refills** Selection of a synchronization date	“You’ll get a phone call from one of our technicians who call you to set everything up [set date] and go from there.” (Exemplar 1) “Then we’ll [the technicians] set their sync date based off the last time that they got that bulk medication… we try to like let the patient know that we’re going to short [fill]. That way we can get it lined up so they can get all their medications at the same time each month.” (Exemplar 7)
**Preparations for medication pick-up and delivery** Initiating contact with patient prior to preparation of prescriptions for pick-up. This communication is essential to ensure that the appropriate medications are refilled and to guide topics for discussion at the appointment. Preparation of the medications for the patient.	“When it [queue] actually pops up in the queue, that’s when the technician will go through and they fill all those medications.” (Exemplar 1) “So, whenever the technicians call for the monthly Med Sync call when they talk to the patient and they kind of get a feel that, you know, the patient does not know what’s going on, or they’re being picky of their medication, they [the technician] tend to triage the call to the pharmacist.” (Exemplar 2) “And so, there’s four workstations, three for technicians and one for pharmacists that are all kind of simultaneously being used to both make synchronization calls as well as fill prescriptions.” (Exemplar 3) “The technicians are supposed to process a certain amount of baskets each day to keep us up ahead of the Med Sync pick up date.” (Exemplar 6) “Mostly I’m [the technician] the main one that does the initial contact and then the initial drop of the prescriptions. So, we attempt to reach out to the patient, let them know that we’re working on their medications for the week.” (Exemplar 7)
**Pick up or delivery of medication and other services** Receipt of medication in person or via delivery. Additional services/interventions may be addressed.	“We’ve had technicians that would offer to deliver their medications too.” (Exemplar 2) “By the point the prescriptions are all filled and ready to go out to the patient. We’ll [technicians] reach out to them again to set up pickup date or delivery date.” (Exemplar 7)
